# Unusual cause of muscle weakness, type II respiratory failure and pulmonary hypertension: a case report of ryanodine receptor type 1(*RYR1)*-related myopathy

**DOI:** 10.1186/s12890-024-03016-7

**Published:** 2024-04-22

**Authors:** Yinong Chen, Shuai Zhang, Xin Lu, Wanmu Xie, Chen Wang, Zhenguo Zhai

**Affiliations:** 1 Peking University China-Japan Friendship School of Clinical Medicine, Beijing, P.R. China; 2 National Center for Respiratory Medicine; State Key Laboratory of Respiratory Health and Multimorbidity; National Clinical Research Center for Respiratory Diseases; Institute of Respiratory Medicine, Chinese Academy of Medical Sciences; Department of Pulmonary and Critical Care Medicine, Center of Respiratory Medicine, China-Japan Friendship Hospital, Beijing, P.R. China; 3 Department of Rheumatology, China-Japan Friendship Hospital, Beijing, P.R. China; 4 Chinese Academy of Medical Sciences, Peking Union Medical College, Beijing, P. R. China; 5 Department of Respiratory Medicine, Capital Medical University, Beijing, P.R. China

**Keywords:** Muscle weakness, Respiratory failure, Pulmonary hypertension, Hypoxia in supine position, *RYR1*, Myopathy

## Abstract

**Background:**

Patients with congenital myopathies may experience respiratory involvement, resulting in restrictive ventilatory dysfunction and respiratory failure. Pulmonary hypertension (PH) associated with this condition has never been reported in congenital ryanodine receptor type 1(*RYR1)*-related myopathy.

**Case presentation:**

A 47-year-old woman was admitted with progressively exacerbated chest tightness and difficulty in neck flexion. She was born prematurely at week 28. Her bilateral lower extremities were edematous and muscle strength was grade IV^−^. Arterial blood gas analysis revealed hypoventilation syndrome and type II respiratory failure, while lung function test showed restrictive ventilation dysfunction, which were both worse in the supine position. PH was confirmed by right heart catheterization (RHC), without evidence of left heart disease, congenital heart disease, or pulmonary artery obstruction. Polysomnography indicated nocturnal hypoventilation. The ultrasound revealed reduced mobility of bilateral diaphragm. The level of creatine kinase was mildly elevated. Magnetic resonance imaging showed myositis of bilateral thigh muscle. Muscle biopsy of the left biceps brachii suggested muscle malnutrition and congenital muscle disease. Gene testing revealed a missense mutation in the *RYR1* gene (exon33 c.C4816T). Finally, she was diagnosed with *RYR1*-related myopathy and received long-term non-invasive ventilation (NIV) treatment. Her symptoms and cardiopulmonary function have been greatly improved after 10 months.

**Conclusions:**

We report a case of *RYR1*-related myopathy exhibiting hypoventilation syndrome, type II respiratory failure and PH associated with restrictive ventilator dysfunction. Pulmonologists should keep congenital myopathies in mind in the differential diagnosis of type II respiratory failure, especially in patients with short stature and muscle weakness.

## Background

Congenital myopathies, a group of rare inherited muscle diseases characterized by abnormalities in the structure of muscle fibers, vary in their clinical presentation, histopathology, and genetic causes [1]. Common clinical features of congenital myopathies include dysmorphic facial features secondary to muscle weakness, abnormal extrinsic eye movements, spinal malformation, malignant hyperthermia, and cardiac or respiratory involvement [1, 2]. Respiratory failure with or without hypercapnia may occur when the diaphragm and other respiratory muscles are affected, resulting in serious complications or even death [2–4]. However, congenital myopathies can be easily misdiagnosed in clinical practice. The main histopathological types of those with respiratory insufficiency include core myopathy, myotubular myopathy, autosomal centronuclear myopathy, congenital fiber-type disproportion myopathy, and myosin storage myopathy [2, 3]. Common genes associated with respiratory involvement include α-skeletal actin (*ACTA1*), nebulin (*NEB*), selenoprotein 1 (*SEPN1*), slow α-tropomyosin (*TPM3*), and ryanodine receptor type 1 (*RYR1*) [1].

Pulmonary hypertension (PH) is a pathophysiological disorder defined by a mean pulmonary arterial pressure (mPAP) > 20 mmHg at rest according to the latest guidelines. PH is divided into five groups based on etiology including chronic respiratory diseases and/or hypoxia [5]. Some patients may experience hypoventilation syndrome and exhibit hypoxemia with or without hypercapnia, which is defined as type II or type I respiratory failure, respectively. One of the causes of hypoventilation is restrictive lung diseases, a common manifestation of congenital myopathies affecting the respiratory muscles [3]. *RYR1*-related myopathies are the most prevalent group of congenital myopathies and have multiple clinical phenotypes, such as symmetric proximal muscle weakness, significant respiratory involvement, King Denborough syndrome, arrhythmias, malignant hyperthermia and so on [6–8]. Up to now, PH has never been reported in cases of congenital *RYR1*-related myopathy, which should be considered as a potential indirect cause in the differential diagnosis of respiratory dysfunction.

Here we report a female patient with *RYR1*-related myopathy who exhibited type II respiratory failure and PH associated with restrictive ventilatory dysfunction.

## Case presentation

A 47-year-old woman was admitted to our hospital with progressively exacerbated chest tightness after activity and echocardiographic suspicion of PH. Her body weight was 50 kg with the height of 150 cm. She was born prematurely at week 28. The patient had no similar family history, but her younger brother was also small and slight in stature. Her chest computed tomography (CT) five years ago showed no significant abnormalities. Four months before this admission, she experienced aggravating dyspnea. Lung function test indicated severe restrictive ventilation dysfunction and reduced diffusion capacity [forced expiratory volume in one second (FEV1) 36%pred, forced vital capacity (FVC) 34%pred, FEV1/FVC 111%, single-breath carbon monoxide diffusing capacity of the lungs (DLCO SB) 47%pred] (Table 1). Re-performed chest CT showed linear opacities, consolidation and nodules. Echocardiography was unavailable at that time. After antibiotics treatment, the symptom of dyspnea was improved while edema in both lower limbs gradually appeared. Meanwhile, she was diagnosed with hypertension (180/100 mmHg at highest) and received antihypertensive agents. Echocardiography two months ago showed mitral and tricuspid regurgitation, which may indicate underlying PH without obvious changes in the structure of the heart. Venous thromboembolism was excluded after CT pulmonary angiography and ultrasonography of the lower extremity veins. However, she had an enlarged main pulmonary artery of 34 mm in diameter. Symptomatic and supportive treatment did not bring significant improvement. One month ago, she was hospitalized again due to aggravated symptoms. The echocardiography indicated massive tricuspid regurgitation, mild right cardiac enlargement and suspicion of PH with an estimated pulmonary arterial systolic pressure (sPAP) of 76 mmHg, lack of signs of left heart disease. Arterial blood gas analysis (ABG) showed a partial pressure of carbon dioxide (PaCO_2_) of 60 mmHg and a partial pressure of oxygen (PaO_2_) of 57 mmHg. She received antibiotics, expectorants, bronchodilator and symptomatic treatment. The edema reduced but the chest tightness did not show significant improvement.

**Table 1:** Arterial blood gas analysis while breathing room air, lung function test and polysomnography measurements

	Four months before	Baseline		Follow-up at 10 months	
	Sitting position	Sitting position	Supine position	Sitting position	Supine position
Arterial blood gas analysis					
pH		7.50	7.49		7.39
PaCO_2_, mmHg		71	72		52
PaO_2_, mmHg		52	38		68
SO_2_, %		89	77		93
FiO_2_		0.21	0.21		0.21
PFR, mmHg		247.62	180.95		323.81
Lung function test					
FEV1, L		0.93	0.37	1.33	
FEV1% (pred)	36.0	43.2	17.4	62.7	
FVC, L		1.12	0.52	1.39	
FVC% (pred)	34.0	44.1	20.7	55.3	
FEV1/FVC, %		83.06	71.26	96.13	
FEV1/FVC% (pred)	111.0	98.4	84.4	113.9	
RV-SB, L		1.42		2.08	
RV-SB % (pred)		97.0		140.4	
TLC-SB, L		2.39		3.48	
TLC-SB % (pred)		58.1		84.7	
DLCO SB% (pred)	47.0	45.8		71.6	
DLCO/VA, mmol/min/kPa/L		1.45		1.70	
DLCO/VA % (pred)		82.4		97.2	
Polysomnography					
Hypopnea index, times per hour			119.6		1.2
Longest duration of hypopnea, seconds			63		51
Average SO_2_ during sleep, %			73		96

*PaCO*_*2*_ Partial pressure of carbon dioxide in arterial blood, *PaO*_*2*_ Partial pressure of oxygen in arterial blood, *SO*_*2*_ Blood oxygen saturation, *FiO*_*2*_ Fraction of inspiration oxygen, *PFR* PaO_2_/FiO_2_ ratio, *FEV1* Forced expiratory volume in one second, *FVC* Forced vital capacity, *RV-SB* Residual volume of single breath, *TLC-SB* Total lung capacity of single breath, *DLCO SB* Single-breath carbon monoxide diffusing capacity of the lungs, *VA* Alveolar ventilation

**Fig. 1:**
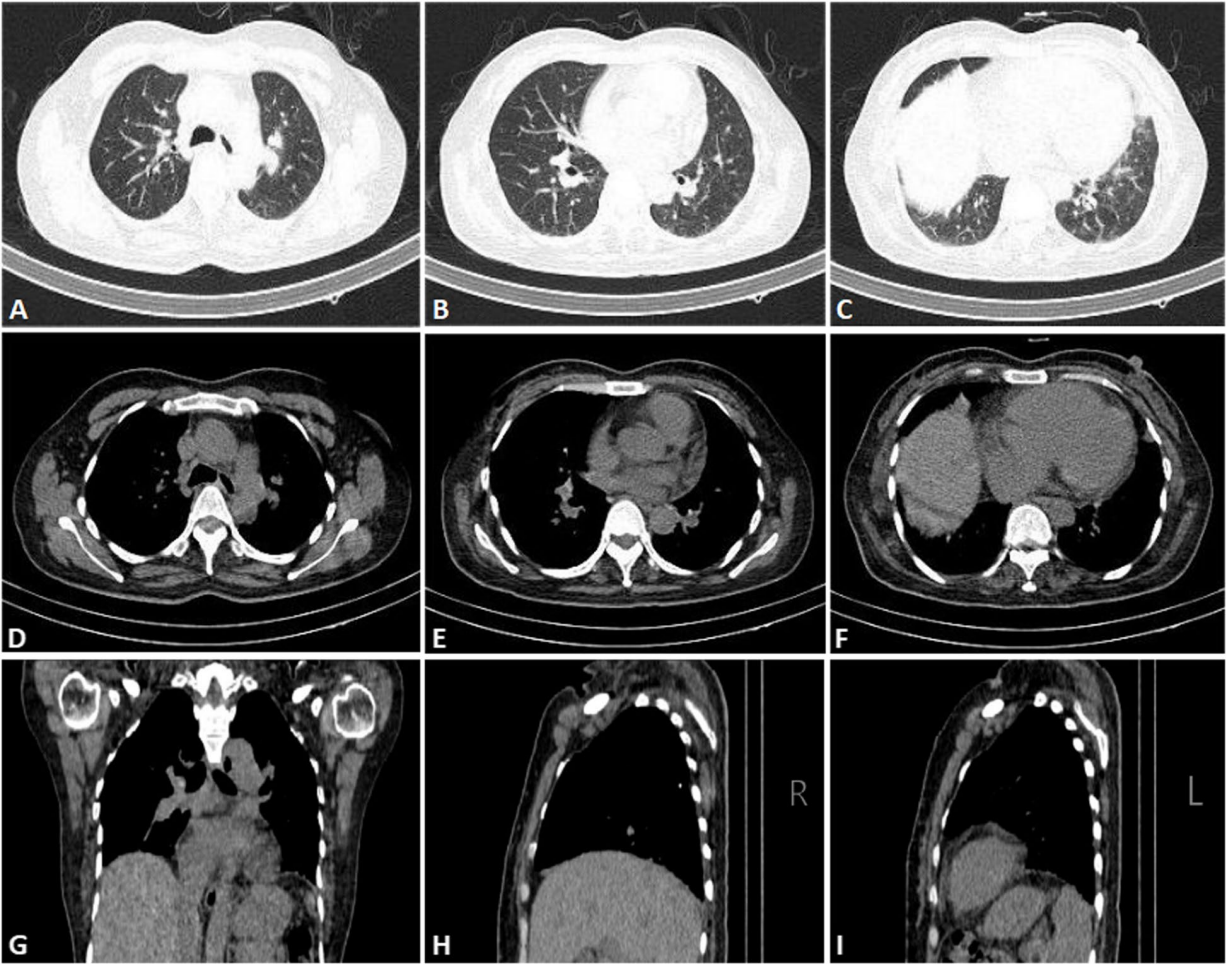
Chest computed tomography of the patient. **A**-**C** lung window; **D**-**I** mediastinal window. Chest computed tomography on admission indicated scattered patchy and linear shadows in bilateral lungs. An elevation of the right diaphragm can be seen in Fig. **C, F** and **G**. Figure **H** and **I** showed the sagittal views of the right and left diaphragm at the same level, respectively

On admission, the patient presented with cyanosis and difficulty in neck flexion. She was afebrile, with a respiratory rate of 20 breaths per minute and a blood pressure of 122/75 mmHg. Heart rate was 109 beats per minute with a loud pulmonic component of the second heart sound. Her bilateral lower extremities were moderately edematous and muscle strength was mildly decreased in grade IV^−^, which defined as being able to engage in activities against light resistance. The admission electrocardiogram showed sinus tachycardia, right axis deviation, and negative T waves in leads V1-V3. The patient presented with hypoventilation, type II respiratory failure and a special phenomenon of hypoxia in the supine position. ABG while breathing room air revealed a PaO_2_ of 38 mmHg in the supine position, but 52 mmHg in the sitting position (Table 1). Lung function test indicated that the residual volume of single breath (RV-SB) was 1.42 L and total lung capacity of single breath (TLC-SB) was 2.39 L. The DLCO/alveolar ventilation (VA) was 1.45 mmol/min/kPa/L, which was 82.4% of the predicted value. Supine FEV1, FVC, FEV1/FVC ratio and the percentage they occupied of the predicted value measurements were all lower than the sitting measurements (Table 1). Lower extremity ultrasound and echocardiography were repeated with similar results to previous examinations. D-dimer in plasma was 1.23 mg/L. Ventilation-perfusion scan was performed and excluded pulmonary embolism, while the simultaneous chest CT indicated scattered patchy and linear shadows in bilateral lungs, and an elevation of the right diaphragm (Fig. 1). RHC indicated that the mPAP was 38 mmHg, pulmonary arterial wedge pressure was 7 mmHg, cardiac output was 4.99 L/min, and pulmonary vascular resistance was 6.21 Wood’s Unit. It revealed severe precapillary pulmonary hypertension, and concurrent acute vasoreactivity testing was negative. Advanced right heart contrast echocardiography showed no shunts. It was less likely to be PH associated with left heart disease, congenital heart disease, or chronic pulmonary artery obstruction. The cause of PH was more likely chronic lung diseases and/or hypoxia. Combining the supine hypoxia phenomenon and restrictive ventilation dysfunction, diaphragmatic ultrasound examination was performed and then revealed reduced mobility of bilateral diaphragm. Specifically, the left diaphragm showed an excursion of 1.0 cm both during quiet breathing and at maximum inspiration, with a diaphragmatic thickening fraction of 26%. On the right side, the diaphragm had an excursion of 1.7 cm during quiet breathing and 1.8 cm at maximum inspiration, with a diaphragmatic thickening fraction of 14%. The red blood cell count, hemoglobin and hematocrit on admission were 5.71 × 10^12^/L, 109 g/L and 44.2%, respectively. In conjunction with anemia-related testing, iron-deficiency anemia was considered and treated. Polysomnography indicated that the patient experienced hypoventilation during nocturnal sleep, with a hypopnea index of 119.6 times per hour. The longest duration of hypopnea lasted 63 s, and the average blood oxygen saturation (SO_2_) during sleep was only 73% (Table 1). Hypoventilation syndrome was definitively diagnosed.

**Fig. 2:**
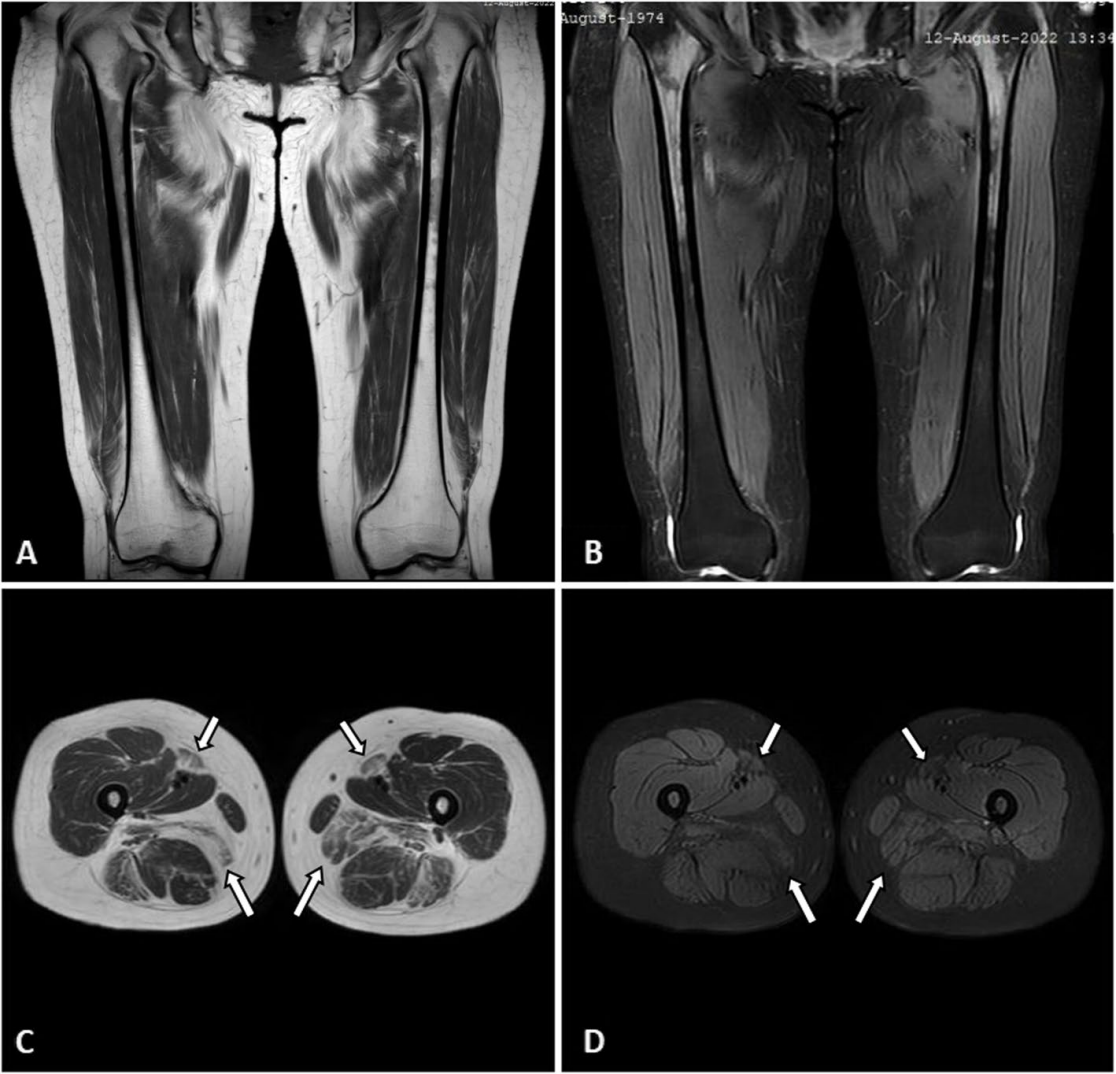
Thigh magnetic resonance imaging of the patient. Magnetic resonance imaging showed bilateral thigh muscle changed consistent with myositis (multiple muscle atrophy and patchy slighted high signal in fat saturated sequence) mainly involved the adductor magnus and sartorius (arrows). **A** T1-weighted non-fat saturated coronal image; **B** T2-weighted non-fat saturated coronal image; **C** synthetic T2-weighted non-fat saturated axial image; **D** synthetic T2-weighted fat saturated axial image

**Fig. 3:**
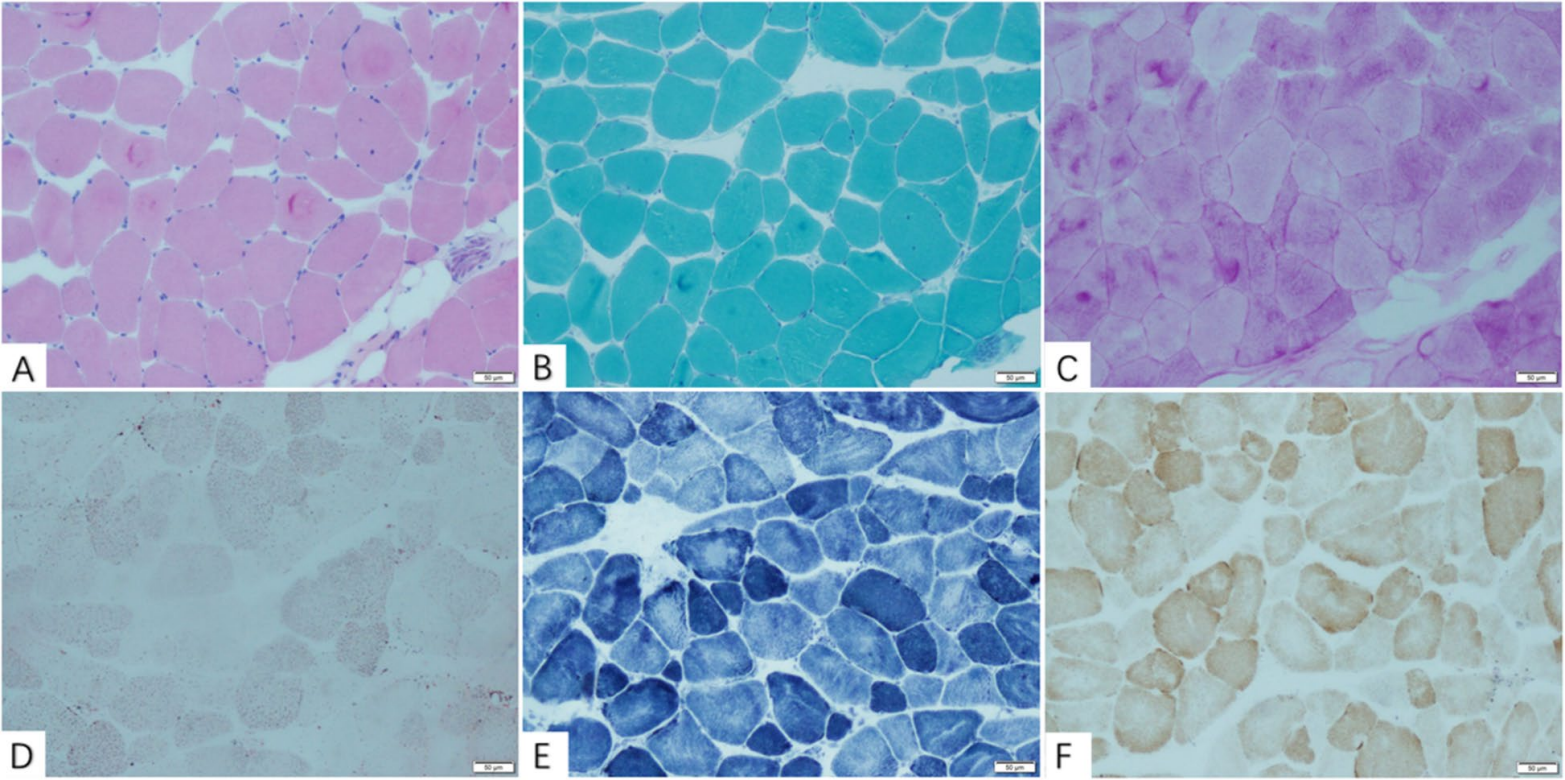
The pathological features of patient with *RYR1* gene mutation. **A** and **B**. H&E and MGT staining × 10: the central core of muscle fiber light stained with surrounded by purple circle; **C** and **D** PAS and ORO staining × 10: no accumulation of glycogen and lipid in muscle fibers; **E** and **F**. NADH-TR and COX staining × 10: the deficiency or light staining of NADH-TR and cytochrome C oxidase in centrally located of muscle fibers

Except for weak positive for anti-TIF1γ antibody and antinuclear antibody (1:80), other immune-related antibody spectra were negative. The creatine kinase (CK) was mildly elevated with a value of 394 U/L. Magnetic resonance imaging (MRI) showed bilateral thigh muscle changed consistent with myositis (Fig. 2). According to the consultation of the rheumatologist, the muscle biopsy was performed on the left biceps brachii and revealed the pathological features of central core disease (Fig. 3).

Thus, we conducted genetic susceptibility gene whole exome sequencing, using the blood sample. The sequencing revealed a missense mutation in the *RYR1* gene (exon33 c.C4816T), which was associated with congenital myopathies. Taking all factors into consideration, the patient was diagnosed with *RYR1*-related myopathy. We initiated non-invasive ventilation (NIV) therapy (Spontaneous-Timed mode, inspiratory positive airway pressure was 14cmH_2_O, expiratory positive airway pressure was 4cmH_2_O, respiratory rate was 14 breaths per minute) to improve her ventilation, while closely monitoring her symptoms, breathing pattern and SO_2_. We used an in-laboratory overnight polysomnogram to titrate NIV in this patient. An oral-nasal mask was chosen for NIV. After regularly using non-invasive positive pressure ventilation at night, her chest tightness and cough significantly improved, so did her exercise tolerance and heart function. In addition to oral diuretics and long-term NIV therapy, we recommended home rehabilitation exercises upon discharge.

After 10 months, she no longer experienced chest tightness or shortness of breath and her muscle strength was better. ABG while breathing room air indicated a higher PaO_2_ of 68 mmHg and a lower PaCO_2_ of 52 mmHg in the supine position. The lung function test suggested an improvement in ventilation function compared to before (Table 1). Repeated echocardiography showed mild tricuspid regurgitation, with a sPAP of 32 mmHg. The red blood cell count, hemoglobin and hematocrit were 4.00 × 10^12^/L, 121 g/L and 36.6%, respectively. Repeated polysomnography indicated improved hypoventilation during nocturnal sleep, with a hypopnea index of only 1.2 times per hour. The longest duration of hypopnea decreased to 51 s, and the average SO_2_ during sleep rose to 96%. We recommended the patient to continue home NIV treatment and rehabilitation, and have regular outpatient follow-up visits.

## Discussion and conclusion

To our knowledge, this is the first report describing a case with *RYR1*-related myopathy presented with hypoventilation syndrome, type II respiratory failure, and PH associated with restrictive ventilator dysfunction. Respiratory muscles, especially the diaphragm, were involved in the congenital myopathy caused by missense mutation in the *RYR1* gene. It impaired the patient's ventilation function and caused hypoventilation syndrome, leading to chronic hypoxemia and hypercapnia, which processed to respiratory failure and resulted in the development of PH eventually.

As for the differential diagnosis process of PH, her medical history, echocardiography and RHC indicated low possibility of PH associated with left or congenital heart disease. The D-dimer test is widely recognized as a valuable biomarker that can rule out venous thromboembolism without the need for costly and time-consuming imaging tests. An approximately fourfold increase in the normal cut-off value of D-dimer in the plasma is associated with a significantly higher risk of pulmonary embolism [9]. This patient had a mild elevated D-dimer and further ventilation-perfusion scan ruled out pulmonary embolism. Meanwhile, her lung function test indicated restrictive ventilation dysfunction. The polysomnography indicated hypoventilation syndrome, while ABG revealed hypercapnia and hypoxemia. There was no evidence for connective tissue disease, HIV infection, portal hypertension or any other possible diseases leading to PH. Thus, we considered that PH was associated with hypoventilation in this patient, which was caused by respiratory muscle dysfunction resulting from myopathy.

PH due to hypoventilation is usually seen in sleep-related breathing disorders [10, 11]. Patients with sleep-related hypoventilation syndromes are often accompanied by hypercapnia and hypoxemia, about half of whom also present with PH and their functional impairment can be improved by NIV [10–12]. Hypoxic and hypercapnic vasoconstriction may play an important pathological role in the reversibility of PH. Meanwhile, sympathetic activation and the elevated viscosity of the blood could also increase the pulmonary vascular resistance, leading to an increase in pulmonary artery pressure [10, 11]. Experiments in rats also found that the PH and increased hematocrit linked to sleep-disordered breathing were a result of chronic intermittent hypercapnic hypoxia [13]. Similar mechanisms may be potentially involved in the development of PH in this patient. Her polysomnography indicated hypoventilation syndrome and ABG revealed hypercapnia and hypoxemia, secondary pulmonary vasoconstriction may explain her reversible PH after NIV. In addition, her follow-up blood routine test showed a decrease in red blood cell count and hematocrit, which may indicate the reduced blood viscosity indirectly. NIV improved her hypoventilation, ultimately improving her cardiopulmonary function.

Congenital myopathies describe a collection of diseases that vary in clinical, histological, and genetic characteristics, primarily impacting muscles. Clinical features, muscle imaging and biopsy, and genetic testing are necessary in the diagnosis [1–3]. The pathogenic genes mainly include dynamin 2 (*DNM2*), myotubularin (*MTM1*), *ACTA1*, slow skeletal β-cardiac myosin (*MYH7*), titin (*TTN*), *NEB*, *SEPN1*, β-tropomyosin (*TPM2*), *TPM3*, amphiphysin 2 (*BIN1*) and *RYR1* [1, 2]. Hypotonia and muscle weakness are the main clinical features [2]. Meanwhile, approximately 64.1% of the individuals exhibited varying degrees of respiratory impairment, with about half of them necessitating nocturnal NIV as a result of respiratory failure [4]. Since diaphragm weakness is one of the main features of myopathy, evaluating diaphragmatic dysfunction is of great importance in these patients. Ultrasonography is a quick and simple method that can be done at the bedside, which showed a strong relationship with the standard respiratory tests commonly used in clinical settings [14]. In the differential diagnosis of respiratory failure, the clinician should pay attention to the patient's medical history and clinical manifestations not only limit to respiratory system, without overlooking some rare but possible causes. Meanwhile, critical examinations such as diaphragmatic ultrasound should be considered in the diagnostic process. Reports of PH indirectly caused by congenital myopathies are relatively rare. PH impacts the quality of life for patients and can be fatal in some circumstances. Therefore, the etiological treatment is crucial for improving the prognosis [5].

Notably, in addition to congenital myopathies, certain diseases can also present with muscle weakness. The differential diagnosis includes neurologic, rheumatologic, endocrine, genetic, medication- or toxin-related, and infectious etiologies [15, 16]. Different neurologic causes can manifest as signs of involvement of either upper or lower motor neurons, or both, with variable patterns of onset and involvement of muscles [15–18]. Creatine kinase levels are generally normal and do not necessitate a muscle biopsy. Weakness caused by inflammatory muscle diseases, with acute or subacute course, mainly affects the proximal muscles. Creatine kinase levels can be moderately or severely elevated, and muscle biopsies commonly show inflammatory infiltration. Patients often experience skin rashes, dysphagia, or other indications of immune system involvement [15, 16, 19]. Endocrine etiologies mainly include adrenal insufficiency, hyperthyroidism, hypothyroidism, Cushing's syndrome and secondary hyperparathyroidism. They also mainly involve the proximal muscles and are accompanied by manifestations of the primary disease [15, 16, 20]. When patients have a history of exposure to drugs (e.g. fluoroquinolones, glucocorticoids, or statins), toxins (e.g. heavy metals, alcohol, or certain recreational drugs), or infections (e.g. viruses or parasites), muscle weakness should be considered as possibly related to these factors after excluding other possible reasons [16, 21–23]. As for genetic causes, most diseases primarily involve proximal muscles. Their patterns of onset often progress gradually. Patients usually have congenital abnormalities in other organs, like the brain, heart, ocular region, skeletal system and so on. Creatine kinase levels are variable, while muscle biopsies often indicate nonspecific myopathic changes, mainly including muscle fiber atrophy, degeneration, and regeneration [15, 16]. When patients exhibit the aforementioned characteristics, it is necessary to consider the possibility of genetic etiologies and to perform genetic testing.

*RYR1*-related myopathy is acknowledged as the most common core myopathy among the congenital myopathies and linked to a broad spectrum of diseases [6, 24]. Although fatigue and weakness were key symptoms of interest among participants with *RYR1*-related myopathy, 2.4% of patients also reported respiratory difficulties [25]. A retrospective cross-sectional study also revealed that 6.8% of patients with *RYR1*-related myopathy had neonatal respiratory involvement and required nocturnal NIV therapy [4]. Muscle biopsy is crucial for diagnosis. Its primary histological subtypes encompass central core disease, multiminicore disease, core-rod myopathy, centronuclear myopathy, and congenital fiber-type disproportion. Regardless of the pathological type, there will be varying degrees of impact of respiratory function, resulting in pulmonary-related complications [6, 26]. A recent study summarized the respiratory features of centronuclear myopathy included *RYR1*-mutated individuals, and found that supine respiratory function measurements of patients were overall lower than sitting measurements [27]. The present case experienced aggravating dyspnea, hypoxia and worse respiratory function in supine position, and hypoventilation during sleep, which were consistent with the characteristics of the disease. Other suggestive clinical features include muscle weakness, hypotonia, myalgia, ophthalmoplegia, bulbar involvement, dysphagia, orthopedic deformities and malignant hyperthermia [6, 24]. The levels of serum CK are often normal or only mildly elevated [2, 7]. When patients exhibit the above characteristics, pulmonologists should consider the possibility of congenital myopathy.

In addition to clinical and histopathologic features, imaging examinations also have diagnostic significance. *RYR1*-related myopathy mainly involves muscle atrophy and intramuscular fatty infiltration [6, 28]. Muscle MRI or ultrasound can detect the patterns of muscle involvement or preservation, while the former is more commonly used and accurate in clinical practice [2, 6, 7]. The mainly involved muscles in the lower limbs include adductor magnus, sartorius, vastus lateralis, vastus intermedius, and vastus medialis, while the rectus femoris, adductor longus, and gracilis are not significantly affected [6]. An effective modified T1-weighted MRI-based algorithm has been proposed to identify and quantify fatty infiltration in patients with *RYR1*-related myopathy [28]. Electromyography (EMG) is less useful in diagnosis given that it can either show normal results or indicate findings similar to myopathy or other neuromuscular disorders in most cases [2]. Thigh MRI of this patient indicated irregular morphology of the bilateral muscles, multiple muscle atrophy, multiple patchy slighted high signals in fat saturated sequence, and blurred fat gaps between muscles. The main muscles involved were the adductor magnus and sartorius, while the adductor longus and gracilis were relatively spared, which consistent with the pattern of muscle involvement and preservation in *RYR1*-related myopathy. Gene testing contributes to the determination of diagnosis and the technology of next-generation sequencing (NGS) makes it more efficient and convenient to use in clinical settings [2, 6].

Main therapies being developed for *RYR1*-related myopathy include drugs that counteract the harmful effects of stress on the cellular environment and the modifications of *RYR1* after protein synthesis, and drugs that directly target *RYR1* or regulate the proteins that influence its functionality [6, 29–31]. Further randomized controlled trials are needed to determine approved therapeutic medications. Moreover, patients with congenital myopathies who presented with nocturnal hypoventilation can benefited from NIV [1–3]. At the same time, we should pay attention to the fact that the presence of sialorrhea and neurobehavioral impairment, and absence of respiratory symptoms can affect negatively of NIV adaptation [32].

There are also some limitations of this report. The patient refused to undergo a follow-up RHC. Thus, we cannot obtain a complete comparison of her hemodynamic parameters after treatment. However, other non-invasive evaluations, such as echocardiography, indeed revealed an improvement in her cardiopulmonary function. Additionally, the underlying mechanisms of PH in this patient cannot be confirmed, further animal experiments will be needed to validate current hypotheses in the future.

In conclusion, we report the first case with *RYR1*-related myopathy who exhibited hypoventilation syndrome, type II respiratory failure, and PH associated with restrictive ventilator dysfunction to the best of our knowledge. When patients exhibit characteristics of muscle weakness, pulmonologists should consider the possibility of myopathies including congenital myopathies. Imaging examinations, muscle biopsy and genetic testing are useful in the differential diagnosis. Meanwhile, attention should be paid to PH related to hypoventilation in clinical practice. NIV is effective and well tolerated in these patients.

## Data Availability

The data that support this case report are available from the corresponding author on reasonable request.

## Abbreviations

*RYR1*: Ryanodine receptor type 1
PH: Pulmonary hypertension
RHC: Right heart catheterization
NIV: Non-invasive ventilation
*ACTA1*: α-skeletal actin
*NEB*: Nebulin
*SEPN1*: Selenoprotein 1
*TPM3*: Slow α-tropomyosin
mPAP: Mean pulmonary arterial pressure
CT: Computed tomography
FEV1: Forced expiratory volume in one second
FVC: Forced vital capacity
RV-SB: Residual volume of single breath
TLC-SB: Total lung capacity of single breath
DLCO SB: Single-breath carbon monoxide diffusing capacity of the lungs
VA: Alveolar ventilation
sPAP: Pulmonary arterial systolic pressure
PaCO_2_: Partial pressure of carbon dioxide in arterial blood
PaO_2_: Partial pressure of oxygen in arterial blood
SO_2_: Blood oxygen saturation
FiO_2_: Fraction of inspiration oxygen
PFR: PaO_2_/FiO_2_ ratio
ABG: Arterial blood gas analysis
SO_2_: Blood oxygen saturation
CK: Creatine kinase
MRI: Magnetic resonance imaging
*DNM2*: Dynamin 2
*MTM1*: Myotubularin
*MYH7*: Slow skeletal β-cardiac myosin
*TTN*: Titin
*TPM2*: β-Tropomyosin
*BIN1*: Amphiphysin 2
EMG: Electromyography
NGS: Next-generation sequencing
